# The Use of Kenaf Fibre as a Natural Anti-Degradant in Recycled High-Density Polyethylene and Natural Rubber-Based Thermoplastic Elastomers

**DOI:** 10.3390/polym15051237

**Published:** 2023-02-28

**Authors:** Nabil Hayeemasae, Cao Xuan Viet, Abdulhakim Masa, Raa Khimi Shuib, Hanafi Ismail, Indra Surya

**Affiliations:** 1 Department of Rubber Technology and Polymer Science, Faculty of Science and Technology, Prince of Songkla University, Pattani Campus, Pattani 94000, Thailand; 2 Department of Polymer Materials, Faculty of Materials Technology, Ho Chi Minh City University of Technology (HCMUT), 286 Ly Thuong Kiet Street, District 10, Ho Chi Minh City 70000, Vietnam; 3 Rubber Engineering & Technology Program, International College, Prince of Songkla University, Hat Yai, Songkhla 90110, Thailand; 4 School of Materials and Mineral Resources Engineering, Universiti Sains Malaysia, Engineering Campus, Nibong Tebal, Penang 14300, Malaysia; 5 Department of Chemical Engineering, Faculty of Engineering, Universitas Sumatera Utara, Medan 20155, Sumatera Utara, Indonesia

**Keywords:** natural rubber, high-density polyethylene, recycling, kenaf fibre, natural weathering

## Abstract

As most plastic materials disintegrate without being properly reused after they are discarded, this present study developed a novel thermoplastic elastomer (TPE) using recycled high-density polyethylene (rHDPE) and natural rubber (NR) with kenaf fibre as a sustainable filler. Apart from being used as filler, this present study aimed to examine the use of kenaf fibre as a natural anti-degradant as well. The results indicated that the tensile strength of the samples was found to have significantly decreased after 6 months of natural weathering and had decreased by a further 30% after 12 months due to the chain scission of the polymeric backbones and the degradation of the kenaf fibre. However, the composites that contained kenaf fibre significantly retained their properties post-natural weathering. In terms of tensile strength and elongation at the break, the addition of only 10 phr of kenaf increased the retention properties by 25% and 5%, respectively. This is noteworthy as kenaf fibre also contains a certain amount of natural anti-degradants. Therefore, as the kenaf fibre improves the weather resistance of composites, plastic manufacturers could use it as either a filler or a natural anti-degradant.

## 1. Introduction

As the global plastic use has steadily increased in the past few decades, it has resulted in the uncontrolled disposal of plastic materials, which adversely affects the environment. The world is producing twice as much plastic waste as 2 decades ago, with the bulk of it ending up in landfill, incinerated, or leaking into the environment, and only 9% being successfully recycled. Plastic consumption has quadrupled over the past 30 years, driven by growth in emerging markets. Global plastics production doubled from 2000 to 2019 to reach 460 million tonnes. Plastics account for 3.4% of global greenhouse gas emissions [1]. As more and more plastic materials are disposed of shortly after use and as plastic materials require years or even decades to degrade, more and more landfills will be needed to accommodate this plastic waste. Therefore, the best way to overcome this issue is to reuse these plastic materials as many times as possible. However, this solution is unsustainable unless these plastic materials are recycled.

A significant portion of plastic waste consists of polyolefins; namely polyethylene (PE) and polypropylene (PP) [2,3]. Therefore, these major types of plastic waste must be effectively recycled. In terms of the production cost, direct recycling may be the best way of recycling plastic waste. However, the value of such plastics can be increased by designing or converting them into new materials. In this regard, thermoplastic elastomers (TPEs) are the material of choice with which to increase the use of recycled plastics. A special class of rubbery material, TPE possesses the properties of both plastic and rubber [4,5]. Apart from their elastomeric behaviour, they are also melt-processable thermoplastics [6]. Therefore, this present study examined TPE produced from natural rubber (NR) and recycled high-density polyethylene (rHDPE).

To extend the use of TPE, it requires the addition of fillers either to improve properties or to further reduce the cost of materials. Recently, there have been many types of fillers incorporated into the TPE regardless of whether they are commercially available or alternative fillers. One source of fillers derived from renewable resources has become a strong competitor to inorganic or commercial fillers. For example, Utrera-Barrios et al. [7] prepared thermoplastic elastomers based on epoxidized natural rubber and polycaprolactone blends in the presence of alginate as a filler. They found that alginate acted as a natural reinforcing filler, increasing the tensile strength of the unfilled rubber from 5.6 MPa to 11.5 MPa without affecting the elongation at the break. Polyethylene (PE) toughened by polyisoprene (IR) was prepared by Ghosh [8]. This polymer matrix was reinforced by lignin. The vulcanization of the polymer matrix with peroxide can improve the mechanical performance of the PE/IR blends, which is further improved with the application of lignin (2 to 5 wt%).

Regardless of the TPE composites, they are normally exposed to natural and artificial environments. This has made chemical, physical, and biological reactions alter their internal properties as well as external appearance [9]. All of these factors induce the natural degradation of polymers [10]. The weathering behaviour of TPE has been extensively studied [11,12,13]. More specifically, TPE predominantly undergoes chain scission and crosslinking during weathering. Photo-oxidation, which occurs when polymeric materials are damaged by exposure to ultraviolet (UV) and visible light, and thermo-oxidation, which occurs due to exposure to thermal and UV light, negatively affect polymeric degradation. Photo-oxidation causes embrittlement and colour changes to occur while thermo-oxidation causes chain degradation via migration, propagation, chain branching, and termination [14].

Much like many other plastic materials, TPE requires the addition of antioxidants to prolong the photo-oxidation process of its polymer. Due to the abundance of commercially available chemical antioxidants, few studies have examined the use of naturally occurring materials. For example, natural fibres, such as banana, sisal, hemp, rice hush, and kenaf to name a few, have not been thoroughly examined for use as natural anti-degradants, although they are low density, relatively cheap, high filling, recyclable, and renewable. Some studies have examined the use of kenaf in composite manufacturing ever since Schneider et al. [15] introduced its use in polymer composites.

It is also a more attractive to substitute environmentally unfriendly inorganic or commercial fillers as it is abundantly available and highly processable. However, as kenaf fibre contains numerous hydroxyl groups on its surface, it is highly sensitive to moisture and, therefore, highly susceptible to degradation via weathering and biodegradation. Furthermore, its cellulose, hemicellulose, lignin, and extractives are also susceptible to photodegradation via UV. However, kenaf fibre contains compounds such as fatty acids, flavonoids, essential oils, and phenolic compounds [16,17]. As such, it exhibits the properties associated with medications, anodynes, anti-inflammatories, aperitifs, aphrodisiacs, and antioxidants [18].

This present study used kenaf fibre as a filler as well as a natural anti-degradant for rHDPE/NR-based TPE. To date, no studies have examined the potential bifunctional use of kenaf fibre in rHDPE/NR composites. This present study examined the tensile, morphological, and thermal properties of the prepared samples pre- and post-natural weathering. Although natural weathering is an extremely lengthy process, it was conducted as it provides the most practical information of in situ composite performance, which is crucial for our understanding of the fundamental degradation process of polymers or their composites, as well as in the prediction of their service lives. As this present study scientifically examines the role of kenaf fibre in rHDPE/NR composites, it is a source of useful information in the preparation of rHDPE/NR-based rubber products.

## 2. Experimental Setup

### 2.1. Materials

The rHDPE, which had a melt flow index of 0.237 g/10 min, as well as the kenaf fibre powder (KP) were manufactured by Zarm Scientific and Supplies Sdn Bhd (Penang, Malaysia), while the light grade standard Malaysian rubber (SMR L) NR was manufactured by Mardec Berhad (Selangor, Malaysia). The KP was obtained from Forest Research Institute Malaysia (FRIM). It was ground in a table type pulverising machine and sieved to obtain a powder that ranged between 32 to 150 µm in size. The chemical composition of KP is listed in Table 1. The rHDPE and KP were then dried in a vacuum oven at 80 °C for 24 h prior to mixing with the NR.

### 2.2. Particle Size Analyser

Particle size analysis was conducted to determine the mean or average particle size of the KP filler. The test was performed at room temperature according to ASTM D 1921 standards in a Malvern Mastersizer Type E. The mean particle size of the KP was its diameter at 50% cumulative size (D_50_).

### 2.3. Preparation of the Blends

The rHDPE/NR blends in the presence of KP were compounded on a HAAKE Rheomix Polydrive R 600/610 mixer at the temperature of 165 °C and a rotor speed of 50 rpm. The content of rHDPE and NR were 70 and 30 parts per 100 polymers (php), while the KP was varied from 0–40 php. To ensure proper mixing, the torque values were recorded at 10 s intervals throughout the mixing process. The rHDPE was first charged in an internal mixer, followed by the NR and the KP. All the samples required approximately 12 min to mix completely. The moulded sheets of the sample were compression-moulded using an electronically heated GT-7014-A30C hydraulic press. They were then preheated at 165 °C for 8 min before they were compressed at a pressure of 100 kg/cm^2^ at 165 °C for 3 min. The samples were then allowed to cool under pressure for a further 2 min.

### 2.4. Natural Weathering

**Figure 1:**
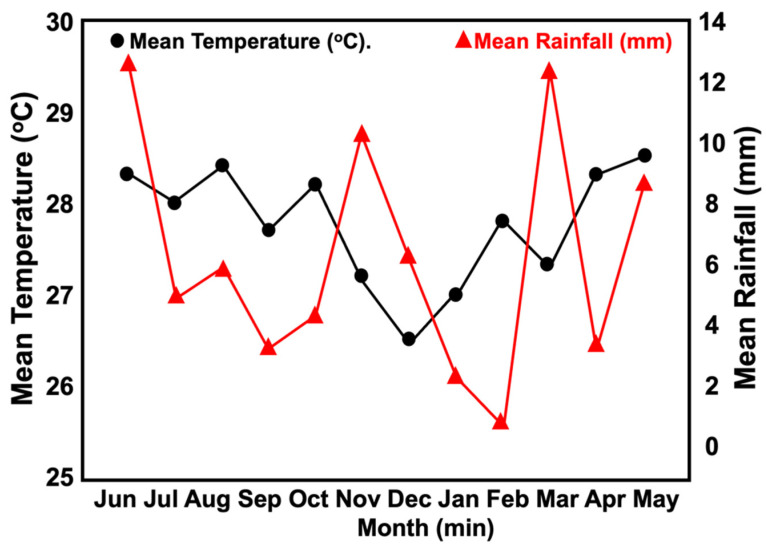
Mean temperature and rainfall between June 2020 to May 2021 in the field area.

The natural weathering tests were conducted according to ASTM D1435 standards in an open area in Penang, Malaysia from June 2020 to May 2021. The Die C dumbbell-shaped samples were cut from moulded sheet and were mounted to a rack at a 45° incline and exposed for 6 (t = 6 m) and 12 (t = 12 m) months, after which time their tensile properties were investigated. Meteorological data was obtained from the nearest meteorology station in Butterworth, Penang [19]. Figure 1 depicts the mean rainfall and temperature during the weathering process. Equation (1) was used to calculate the tensile properties retention of the rubber blends which, in turn, indicated their heat resistance properties:

Retention (%) = (value after aging/value before aging) × 10(1)

### 2.5. Tensile Properties

An Instron 3366 universal testing machine (Instron Corporation, Norwood, MA, USA) was used to conduct tensile tests according to ASTM D412 standards. The Die C-shaped samples were stretched at a crosshead speed of 50 mm/min. The data obtained from this test indicated the tensile strength, modulus, and elongation at the break of the samples. The tensile strength and elongation at the break are the ultimate stress and strain, respectively, while the tensile modulus is measured from the linear slope of the stress-strain curve.

### 2.6. Fourier Transform Infra Spectroscopy (FTIR)

A PerkinElmer 2000 Fourier transform infra spectroscope (FTIR) was used to determine the chemical functionalities of the KP and the samples pre- and post-natural weathering. All the spectra were obtained using attenuated total reflectance (ATR) and recorded in the transmittance mode from 4000 to 500 cm^−1^ with a resolution of 4 cm^−1^.

### 2.7. Scanning Electron Microscopy (SEM)

A Supra-35VP field emission scanning electron microscope (SEM) was used to examine the tensile fractured samples to determine the morphology of the KP and the samples pre- and post-natural weathering. A Bio-Rad Polaron Division device was first used to sputter-coat the samples with gold/palladium prior to scanning to prevent electrostatic charging during imaging. The SEM images were also used to determine the diameter and fibre length of the KP distribution to obtain additional information regarding the filler characteristics. Image Pro Plus 6 was used to analyse 160 fibres seen in the SEM images. The aspect ratio of the kenaf fibre was determined by dividing the average length by the average diameter.

### 2.8. Thermogravimetric Analysis (TGA)

A PerkinElmer Pyris 6 thermogravimetric analyser (TGA) was used to thermogravimetrically analyse the samples. The samples were heated from 30 °C to 600 °C at a rate of 10 °C/min in a nitrogen atmosphere and periodically scanned.

### 2.9. Differential Scanning Calorimetry (DSC)

A PerkinElmer DSC-7 analyser was used to measure the melting and crystallisation characteristics of the samples. The thermal history was removed by annealing the sample at the temperature of 140 °C, which is higher than the melting temperature (T_m_) of rHDPE, and then cooled it down. Next, endothermic and exothermic conditions were performed. The samples were heated from 30 °C to 200 °C at a heating rate of 10 °C/min in a nitrogen atmosphere. The samples were cooled down at the same parameters. Equations (2) and (3) were used to determine the crystallinity of the (*X_com_*) composite and the rHDPE fraction (*X_rHDPE_*) pre- and post-surface treatment.

(2) $$X_{com}=\frac{\Delta H_{f}}{\Delta H_{f}^{0}}\times 100$$

(3) $$X_{rHDPE}=\frac{X_{c}}{W_{f(rHDPE)}}\times 100$$

where Δ*H_f_* was the heat of fusion of the sample, Δ*H*^0^*_f_* (PE) = 293 J/g [20] was used for the 100% crystalline rHDPE homopolymer, and *W_f_*_(*rHDPE*)_ was the weight fraction of rHDPE in the composite.

## 3. Results and Discussion

### 3.1. Characterization of KP

**Figure 2:**
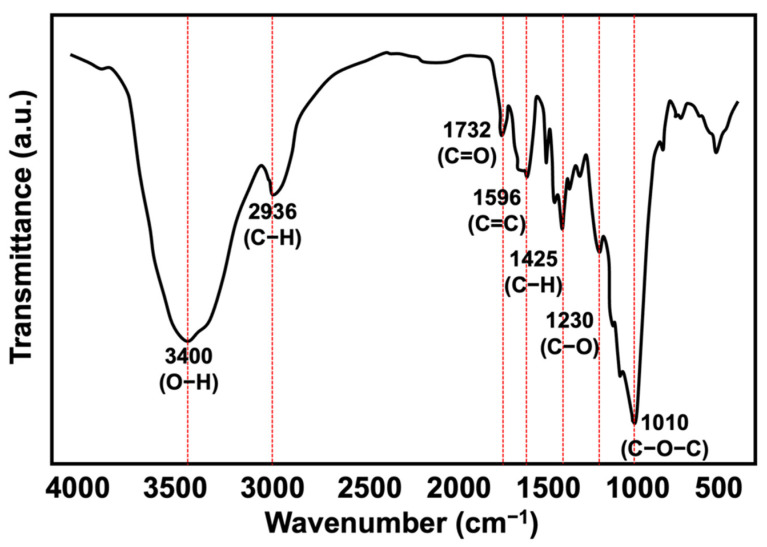
FTIR spectrum of KP.

The physical characteristics of the KP, such as its fibre diameter, length, shape, and particle size, to name a few, were determined prior to compounding with the rHDPE/NR blend matrix as these parameters may influence the mechanical properties of the composites. Although the chemical properties of the fillers generally do not change during the melt mixing processes, their physical characteristics can change. As such, a particle size analyser, FTIR, SEM, and TGA were used to examine the KP. Figure 2 depicts the FTIR spectrum of the KP. The observed spectrum could be classified into a few regions, as an O-H stretching band was observed at the wavenumber of 3400 cm^−1^, C-H stretching was observer at the wavenumber of 2936 cm^−1^, and CH_2_ symmetric bending of C=C in an aromatic group was observed at the wavenumber of 1425 cm^−1^, while carbonyl, alkene, and ether linkages were related to the bands observed at the wavenumbers of 1732, 1596, 1230, and 1010 cm^−1^, respectively. These FTIR results indicated the presence of hemicelluloses, lignin, cellulose, and other components in the chemical composition of the kenaf core fibre.

**Figure 3:**
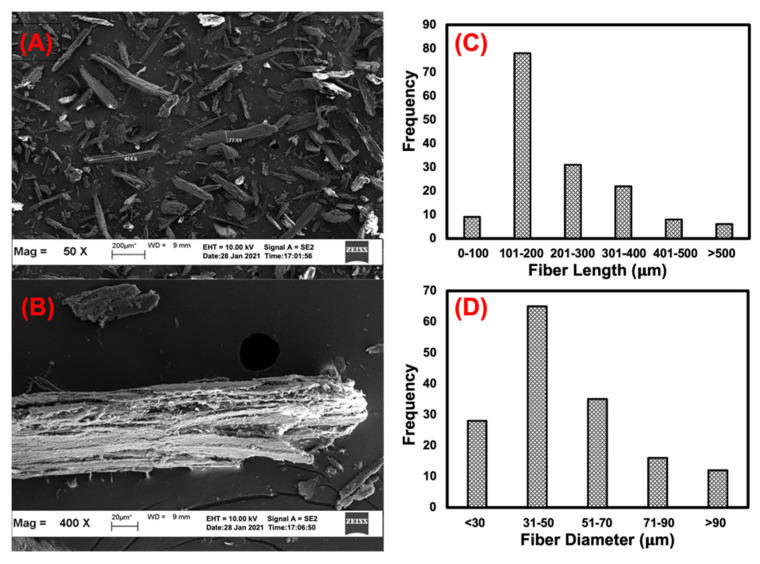
SEM images of KP (**A**,**B**) and its fibre length and fibre diameter distribution (**C**,**D**).

Figure 3A,B depicts the SEM micrographs of the KP. A mixture of short KP fibres and KP particulates of significantly different sizes were observed. It also contained irregularly shaped particles, which could be due to the grinding process. As seen in Figure 3B, a magnified view of the KP fibres showed an uneven and rough surface with some debris. The shape of the filler was well-described by its aspect ratio, which was the length by the diameter of a particle. A total of 160 fibres were investigated to determine the length and diameter of the KP. Figure 3C,D depicts the fibre lengths and diameters, respectively. Most of the fibres were between 101 to 200 µm in length and 31 to 50 µm in diameter. The median particle size was later confirmed by particle size distribution.

The term “particle size” can be misleading and misunderstood, as a small filler sample contains many particles of different sizes and shapes. Therefore, the term “particle size distribution” is more suitable for this present study. Table 2 provides the sizes and the span factor of the particles. The span factor is a measure of the width of the particle size distribution. Inorganic fillers such as talc and silica commonly have a lower span factor [21]. This narrow particle size distribution indicates that the particle sizes are uniformly distributed. Kenaf core powder also contains a mixture of particle shapes. As such, it should be further characterised according to its aspect ratio. Helium pycnometer was used to determine the density of the KP, which was 1.48 g/cm^3^. This corresponds to the natural fibre density reported by extant studies, which ranged between 0.9 to 1.6 g/cm^3^ [22].

**Figure 4:**
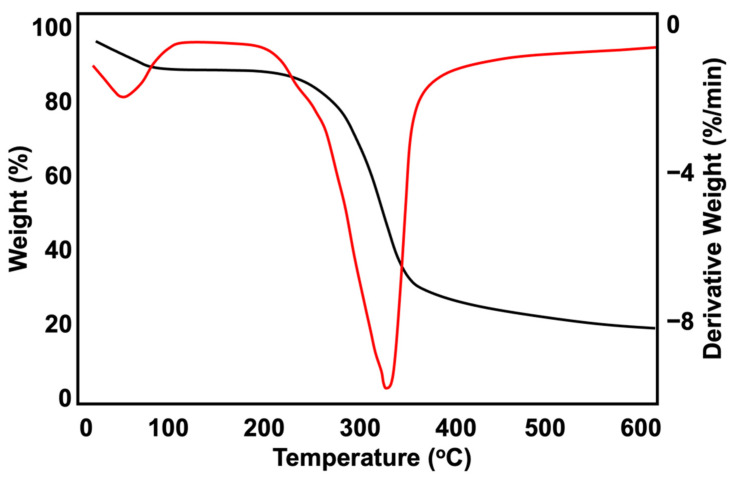
TGA (black) and DTG (red) profiles of KP.

When developing natural fibre composites, it is crucial to determine the thermal degradation of the natural fibre, as it strongly affects the maximum temperature that can be used to process the composites [23]. As such, a TGA test was conducted in a nitrogen atmosphere with temperatures ranging between 30 to 600 °C. The thermal behaviour of lignocellulosic materials primarily depends on their chemical compositions, structures, and degrees of crystallisation. Figure 4 depicts the TGA results of the kenaf core fibre. The KP thermally decomposed rapidly between 200 and 400 °C. The first peak on the derivative thermogravimetric (DTG) curve occurred at 63 °C due to moisture evaporation [24], while the small shoulder seen at 255 °C and the strong sharp peak at 324 °C were due to the decomposition of the hemicellulose and α-cellulose, respectively [25].

### 3.2. Natural Weathering

**Figure 5:**
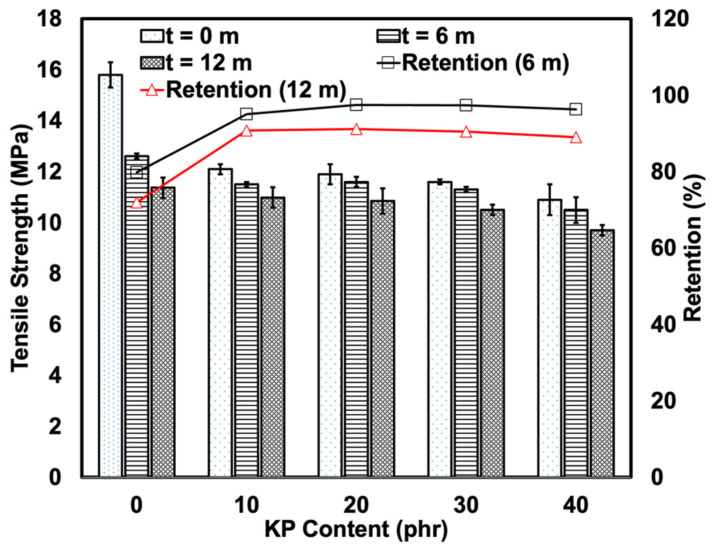
Tensile strength and retention of KP-filled rHDPE/NR blends before and after 6 and 12 months of natural weathering.

Figure 5 depicts the change in tensile strength, elongation at break, and tensile modulus of the samples pre- and post-natural weathering. Prior to the natural weathering process, the tensile strength of the KP-filled rHDPE/NR composites decreased gradually as the KP content increased. The weak interfacial bond between the hydrophilic lignocellulosic filler and the hydrophobic polymer matrices prevented stress propagation. As such, the composites could not elongate and break when the internal stress increased at the interface of the filler and the matrix, resulting in lower tensile strength. In terms of size, the KP was larger than other reinforcing fillers, such as carbon black (~8–100 nm) or silica (~50–1000 nm) [26]. As such, the KP failed to reinforce the polymer matrices as a filler. The poor stress transfer properties of the composites could also be due to the irregular morphology of the KP, which hindered KP orientation during the tensile test and deteriorated the tensile strength.

The tensile strength of the samples decreased post-severe weathering. The tensile strength of all the weathered samples decreased post-weathering. In the samples that contained a fixed amount of KP, the tensile strength most likely decreased as the constituents of the polymer degraded, due to exposure to environmental factors, such as moisture, sunlight, and chemical pollutants, to name a few. The tensile strength of the samples was found to have significantly decreased after 6 months of natural weathering and had decreased by a further 30% at 12 months. This could be due to the degradation of the polymer itself via photo-oxidation, which results in the scission of polymer chains when exposed to UV. The scission of larger molecular chains creates several shorter chains, which decreases entanglement and tensile strength. Furthermore, NR is more prone to degradation and degradation occurs more rapidly in NR than in rHDPE as it contains unsaturated main chains [27].

**Figure 6:**
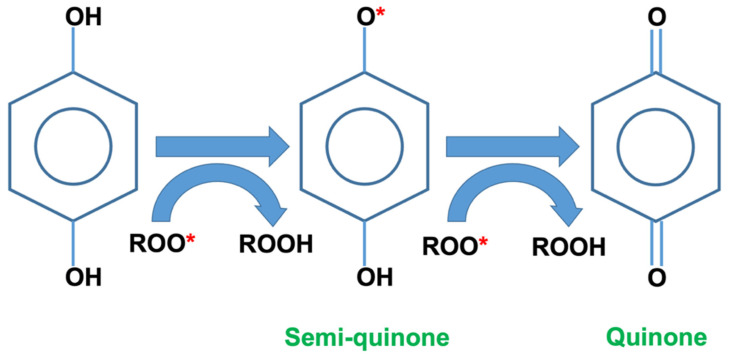
Schematic illustration of the anti-degradant function of phenolic-contained lignin (* is the radical).

It is noteworthy that the KP-filled composites retained their tensile strength better than unfilled composites. The KP may have formed interparticle cavities that acted as ‘‘dampeners’’ that absorbed the energy of propagating cracks before they reached other parts, thereby minimising their deleterious effects [28]. The KP also slowed the photo-oxidation of the polymer matrix. According to Abu-Sharkh and Hamid [29], as kenaf fibres contain lignin, a natural antioxidant that stabilises the fibre, they stabilise the composites [30]. Lignin contains highly aromatic and complex phenolic structures that are amorphous and cross-linked [31]. As seen in Figure 2, the KP had a high phenolic content as evidenced by the strong O-H stretching band. Phenolic compounds could increase the durability of plastics in natural weather. Figure 6 depicts the mechanism of action of phenolic as an antioxidant via free radical scavenging and metal chelation. It also inhibits the production of reactive oxygen species by scavenging the free radicals that are created by chain scission [32].

The dark colour of the surface of the fibres also functions as a protective layer that prevents UV penetration into the samples. A higher KP content resulted in the same retention of tensile strength. This may be due to the degradation of the KP or fibre components, such as lignin, hemicelluloses, and cellulose. As the fibre components are considered polymeric materials, degradation may occur, which limits the efficacy of the KP as a natural anti-degradant as some of the physical, chemical, and biological properties of the lignocellulosic material decreases [31]. As seen in Figure 1, the KP degradation did not appear too severe, as the tested temperatures only ranged between 26 to 29 °C, which was not high enough to severely degrade the KP and the polymer matrices. Furthermore, as seen in Figure 4, which depicts the result of the TG profiles, the KP only began to slightly degrade at 63 °C, which was significantly higher than the mean temperature of natural weather conditions.

**Figure 7:**
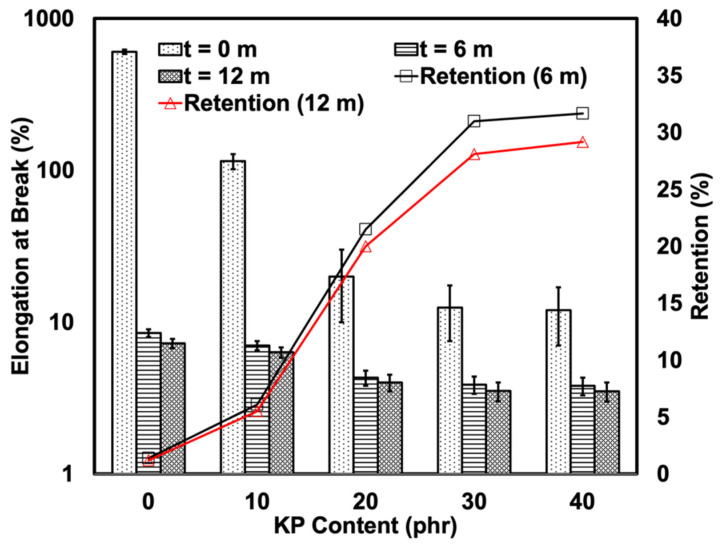
Elongation at break and retention of KP-filled rHDPE/NR blends before and after 6 and 12 months of natural weathering.

Figure 7 depicts the elongation at the break of the composites pre- and post-natural weathering. As KP is rigid in nature, the KP-filled rHDPE/NR composites exhibited significantly lower elongation at the break as the KP decreased the flexibility of the composites. The reduction in elongation at the break was most evident at KP loadings of 40 phr. The significantly lower elongation at the break of the KP-filled rHDPE/NR composites could also be due to the weak adhesion between the polymer matrices and the KP, which made them more prone to fracture. The elongation at the break of all the samples significantly decreased post-6 and 12 months of exposure to natural weather. As previously mentioned, this could be attributed to the breakdown of the polymer chains and the disentanglement of the matrix in the samples with a fixed KP content post-exposure. According to Leong et al. [28], the embrittlement of the composites could occur when they hydroperoxides and carbonyl groups that are created during photo-oxidation combine with the aromatic compounds accumulated on the surface of samples. The unfilled rHDPE/NR composite exhibited the most apparent decrease in elongation at the break. Pre-natural weathering, its deformability was superior to that of its filled counterparts. However, its elongation at the break decreased by 98% post-natural weathering. Meanwhile, the elongation at the break of the filled composites remained almost the same as its unfilled counterparts, regardless of being after 6 or 12 months of natural weathering. This was more evident in the retention of their properties which increased up to 32%. Therefore, KP could act as a protective layer that inhibits the degradation of the polymer matrix.

**Figure 8:**
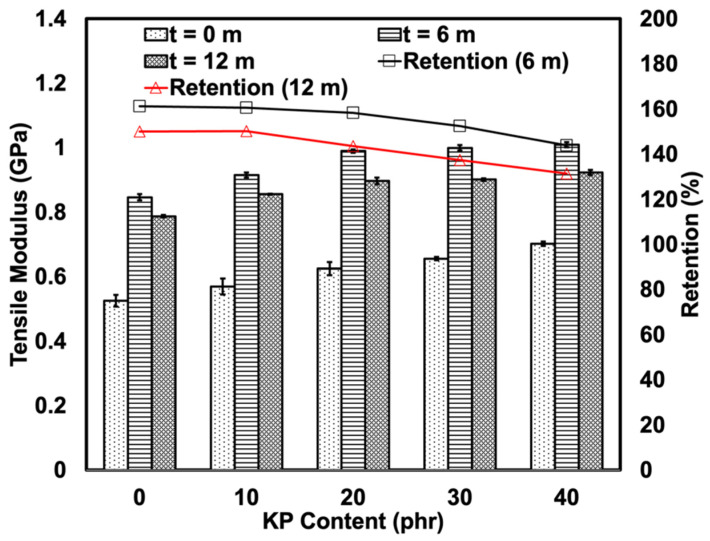
Tensile modulus and retention of KP-filled rHDPE/NR blends before and after 6 and 12 months of natural weathering.

Figure 8 depicts the tensile modulus of the composites pre- and post-thermal aging. The addition of KP, which is a rigid filler, was expected to increase the tensile modulus of the soft matrix. The tensile moduli of the weathered composites were higher than that of the non-weathered composites. This increase in tensile modulus could be due to two factors, depending on the matrix of the composite. As for the rHDPE, the harder effect may be attributed to an increase in crystallinity and stiffness due to photo-oxidation. When polyethylene is weathered, it results in a chain scission that produces shorter chains that are more mobile and can crystallise more readily [32]. Meanwhile, in NR, the chain scission of molecular chains could generate radicals that combine and form newer bonds that may increase the stiffness of the NR. However, longer exposure to natural weathering causes surface cracks to develop and enables chemical degradation and water absorption to occur. It is noteworthy that natural weathering did not alter the tensile modulus of the KP-filled rHDPE/NR composites as the KP may have absorbed the moisture. This may have softened the KP and decreased the overall stiffness of the KP-filled rHDPE/NR composites.

### 3.3. Surface Morphology

**Figure 9:**
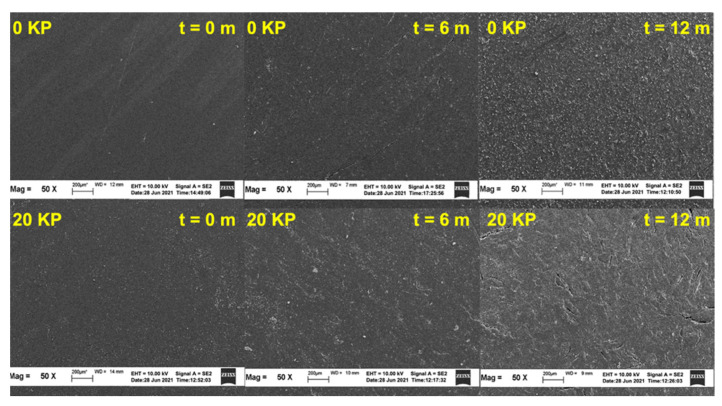
SEM micrographs at 50× magnification of KP-filled rHDPE/NR blends before and after 6 and 12 months of natural weathering.

**Figure 10:**
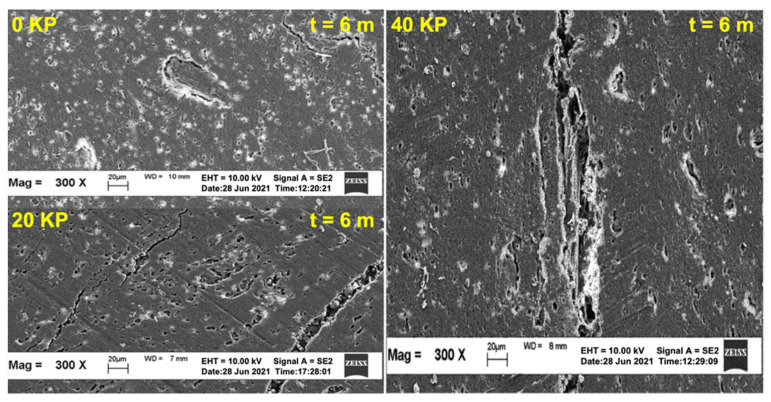
SEM micrographs at 300× magnification of KP-filled rHDPE/NR blends before and after 6 and 12 months of natural weathering.

Figure 9 and Figure 10 depict the SEM images of the tensile-fractured samples pre- and post-6 and 12 months of natural weathering. As seen in Figure 9, the SEM micrographs indicate that the surface of the non-weathered samples was relatively smooth and crack-free. Although some defects such as scratches and dust were observed due to mould covers, they should not have affected the tensile properties of the samples. However, some cracks and impurities (dust) were observed on the surfaces of the KP-filled rHDPE/NR composites. Although these defects were present on the surfaces of all the samples after extended weathering, the severity of the defects differed from one another. Post-natural weathering, the SEM images deteriorated severely after 6 and 12 months as several long and large cracks appeared on the surface. These cracks formed due to UV exposure, which increased the crystallinity of the surface and caused shrinkages [28]. Thermal stress could also have contributed to the formation of these cracks [33].

As seen in the SEM images in Figure 10, KP-filled rHDPE/NR composites better retained their properties than the unfilled composites. They also contained very few and smaller cracks with no preferential crack propagation direction. As such, these surfaces were not severely damaged, and the cracks ended at this site. Therefore, the tensile failure of these composites was determined using their interior properties. This may be the reason KP-filled rHDPE/NR composites better retained their tensile properties than the unfilled composite. However, when the composites were loaded with 40 phr of KP, more and larger cracks were observed on the surface. A closer examination of the weathered surface revealed some micro-cracks, holes, and fibre damage. Furthermore, the KP-filled rHDPE/NR composites had smaller-sized holes and surface cracks also appeared at the matrix-fibre interface. The surface cracks may have been caused by the swelling of the embedded fibres due to moisture absorption from the environment, which could also facilitate the formation of severe cracks in the composites.

### 3.4. Changes in Functionalities after Natural Weathering

**Figure 11:**
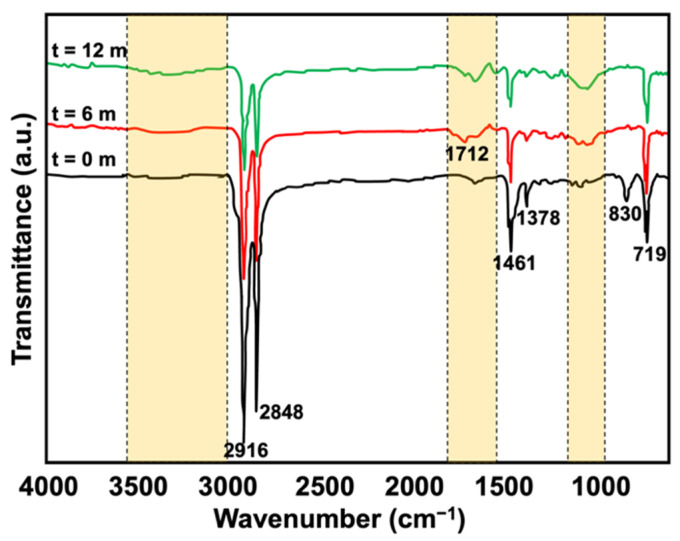
FTIR spectra of KP-filled rHDPE/NR blends before and after 6 and 12 months of natural weathering.

**Figure 12:**
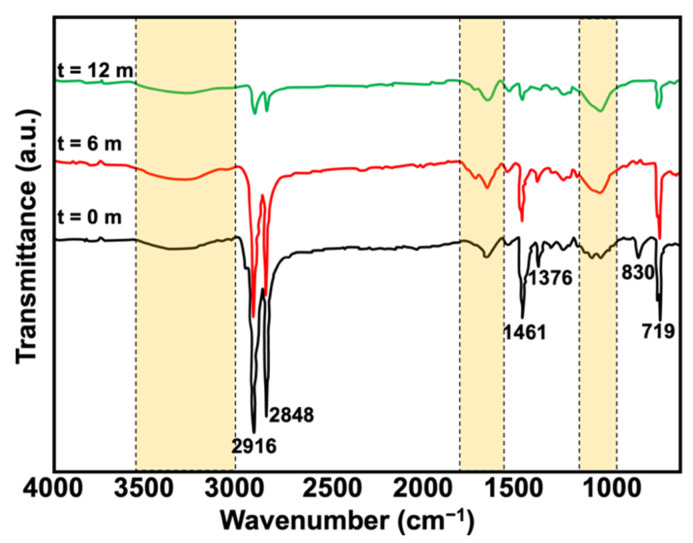
FTIR spectra of rHDPE/NR blends filled with 20 phr of KP before and after 6 and 12 months of natural weathering.

Figure 11 and Figure 12 depicts the FTIR spectra of the composites pre- and post-6 and 12 months of natural weathering. As seen, some peaks disappeared. The peak seen at approximately 1712 cm^−1^ (t = 6 and t = 12) indicated the presence of the carbonyl group due to the formation of carboxylic, aldehyde, and ketone species [34]. The spectra also showed an increase in the intensity of the vinyl groups (800–1000 cm^−1^), which very commonly occurs when polymeric materials degrade. Such functionalities form when chain scission occurs in the presence of oxygen and moisture. The intensity of the peaks seen at 2916, 2848, 1461, and 1376 cm^−1^ had decreased, while the peaks seen at 830 cm^−1^ had disappeared. This was simply due to the formation changing from a pure hydrocarbon structure to other functionalities. The hydroxyl groups were also noted post-weathering in the region of 3400 to 3600 cm^−1^, with broader peaks and stronger shoulders. This confirmed that the weathered samples underwent photo-oxidation as the degradation occurred in the air atmosphere in the presence of oxygen and heat. This increased the reactive oxygen species and could impart several functionalities, where hydroxyl is one of the dominant peaks in the FTIR spectrum of a polymer post-degradation.

### 3.5. Differential Scanning Calorimetry

**Figure 13:**
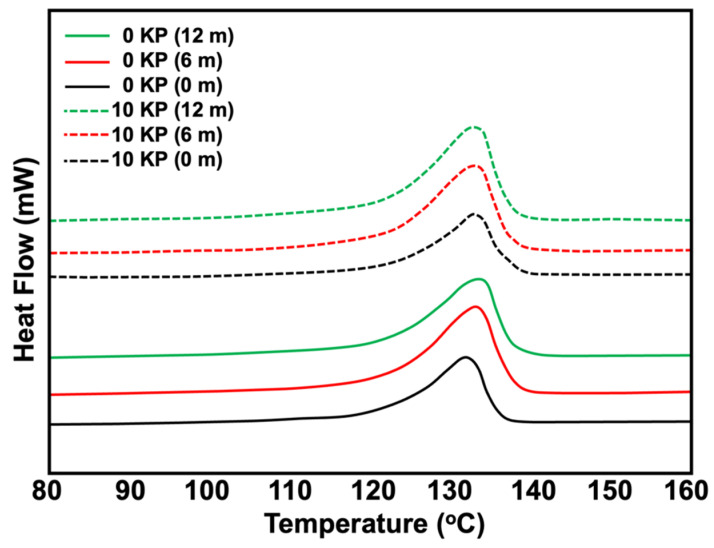
DSC thermogram of KP-filled rHDPE/NR blends before and after 6 and 12 months of natural weathering.

Figure 13 depicts the differential scanning calorimetry (DSC) thermograms of the composites pre-and post-6 and 12 months of natural weathering. A DSC was conducted to evaluate the crystallinity and other behaviours of the composites post-natural weathering. The melting behaviour of the weathered samples was like that of the non-weathered samples as their DSC curves did not differ significantly. This suggests that the crystalline phase did not change and that the amorphous regions underwent degradation. As polyethylene is a semi-crystalline polymer, the packing of its crystalline phase is much tighter than that of its amorphous phase and is, therefore, impermeable to oxygen [35]. As such, degradation may have occurred predominantly in the amorphous region of the rHDPE or NR phases and was governed by the oxygen diffusion in these regions.

Table 3 provides a more detailed summary of the DSC results. The melting temperature of the rHDPE in the composites did not significantly change after post-natural weathering. The melting temperature (T_m_) of the composites was slightly lower than that of the non-weathered sample due to oxidative reactions on the crystal surfaces, which increases the free surface energy of the crystals [28]. In addition, the T_m_ of the weather rHDPE/NR composites was more or less the same as the exposure time increased. The crystallinity of the weathered samples increased post-6 months of natural weathering. This may be due to the rearrangement of the amorphous phase after chain scission due to photo-oxidation [35]. Bhateja et al. [36] reported that the increase in crystallinity during the first period of degradation was due to the severed tie molecules traversing the amorphous regions, allowing the existing crystalline lamellae to increase in perfection and new lamellae to grow. Meanwhile, a longer exposure time (12 months) caused a slight decrease in crystallinity. This may be because the chain scission effect predominantly occurred only at the initial stages. When the exposure time increased, the rHDPE chemically degraded and formed carbonyls and hydroperoxides. As impure groups attached to the molecular segments, they could no longer fit into the crystal lattice, thereby hindering the crystallisation process [37]. The statistical analysis of the DSC data before and after natural weathering was also performed with the SigmaPlot^®^ program (SPSS Inc., Chicago, IL, USA). The results from KP content and weathering time were used to construct the statistical difference of the samples ZnO. The differences among the treatment groups were examined by using a two-way analysis of variance followed by a Tukey comparisons test. The differences were considered to be statistically significant at *p-*values less than 0.05. Table 4 shows the p-value obtained from statistical analysis. The results showed a statistically significant difference in T_m_, heat of fusion, and crystallinity when considering the KP content (*p* < 0.05). However, an insignificant difference was observed when considering the weathering times regardless of 6 or 12 months. This clearly supported the evidence from DSC outputs of the blends.

## 4. Conclusions

The present study examined the use of KP as a filler and natural anti-degradant for rHDPE/NR-based TPE. The tensile strength and elongation at the break of the composites decreased post-weathering due to polymeric chain scission. The tensile strength of the samples was found to have significantly decreased after 6 months of natural weathering and had decreased by a further 30% at 12 months. However, it is noteworthy that the properties retention inversely increased as the natural weathering duration increased. This was most evident in the retention of tensile strength and the elongation at break properties, which increased by 25%. Therefore, the composites were weather-resistant. Based on the results, the addition of 20 phr of kenaf fibre to rHDP*E*/NR-based TPE was best suited when a product requires mechanical properties and weather resistance. Therefore, plastic manufacturers should take kenaf fibre into consideration as either a filler or natural anti-degradant as it extends the use and decreases the cost of manufacturing rHDPE/NR-based TPE.

## Figures and Tables

**Table 1:** Chemical composition of KP.

Chemical Component	Amount (%)
Cellulose	46 ± 0.5
Hemicellulose	33 ± 2.0
Lignin	20 ± 1.0
Extractives	2.2 ± 0.5
Ash	3.0 ± 0.4

**Table 2:** The 10th percentile (d_10_), 90th percentile (d_90_), and median percentile (d_50_) of particle size distribution, span factor, surface area, and density of KP.

Filler	d_10_ (µm) ^a^	d_50_ (µm) ^a^	d_90_ (µm) ^a^	Span Factor	Surface Area (m^2^/g) ^a^	Density (g/cm^3^) ^b^
KP	8.4	43.8	105.2	2.2	0.383	1.48

a = Malvern Mastersizer Type E; b = AccuPyc II 1340 Pycnometer.

**Table 3:** DSC outputs of KP-filled rHDPE/NR blends before and after 6 and 12 months of natural weathering.

Sample	Before Weathering			Weathering for 6 Months			Weathering for 12 Months		
	T_m_ (°C)	∆*H_f_*_(*com*)_ (J/g)	*X_rHDPE_* (%)	T_m_ (°C)	∆*H_f_*_(*com*)_ (J/g)	*X_rHDPE_* (%)	T_m_ (°C)	∆*H_f_*_(*com*)_ (J/g)	*X_rHDPE_* (%)
0 KP	131.95	104.61	51	133.20	119.67	58.35	133.39	120.00	58.51
10 KP	132.95	93.35	50.07	132.84	117.32	62.92	132.87	116.21	62.33
20 KP	132.58	75.49	51.52	132.78	97.06	66.25	132.33	79.67	54.38

**Table 4:** The *p*-value obtained from statistical analysis of KP-filled rHDPE/NR blends before and after 6 and 12 months of natural weathering.

Source of Variation	T_m_ (^o^C)		∆*H_f_*_(*com*)_ (J/g)		*X_rHDPE_* (%)	
	*p*-Value	*p* < 0.05	*p*-Value	*p* < 0.05	*p*-Value	*p* < 0.05
KP Content	0.552	No	0.031	Yes	0.043	Yes
Weathering Time	0.708	No	0.006	Yes	0.73	No

## Data Availability

The data presented in this study are available on request from the corresponding author.
